# Deferiprone-Gallium-Protoporphyrin Chitogel Decreases *Pseudomonas aeruginosa* Biofilm Infection without Impairing Wound Healing

**DOI:** 10.3390/ma17040793

**Published:** 2024-02-07

**Authors:** Tahlia L. Kennewell, Hanif Haidari, Suzanne Mashtoub, Gordon S. Howarth, Catherine Bennett, Clare M. Cooksley, Peter John Wormald, Allison J. Cowin, Sarah Vreugde, Zlatko Kopecki

**Affiliations:** 1 Future Industries Institute, University of South Australia, Mawson Lakes, SA 5095, Australia; tahlia.kennewell@unisa.edu.au (T.L.K.); hanif.haidari@unisa.edu.au (H.H.); allison.cowin@unisa.edu.au (A.J.C.); 2 School of Biomedicine, The University of Adelaide, Adelaide, SA 5005, Australia; suzanne.mashtoub@adelaide.edu.au; 3 Department of Gastroenterology, Women’s and Children’s Hospital, North Adelaide, SA 5006, Australia; 4 School of Animal and Veterinary Sciences, The University of Adelaide, Roseworthy, SA 5371, Australia; gordon.howarth@adelaide.edu.au; 5 Adelaide Medical School, The University of Adelaide, Adelaide, SA 5005, Australia; catherine.bennett@jonesradiology.com.au (C.B.); clare.cooksley@adelaide.edu.au (C.M.C.); peterj.wormald@adelaide.edu.au (P.J.W.); sarah.vreugde@adelaide.edu.au (S.V.); 6 Department of Surgery Otolaryngology Head and Neck Surgery, Basil Hetzel Institute for Translational Health Research, Central Adelaide Local Health Network, Woodville South, SA 5011, Australia

**Keywords:** *Pseudomonas aeruginosa*, wound infection, biofilms

## Abstract

*Pseudomonas aeruginosa* is one of the most common pathogens encountered in clinical wound infections. Clinical studies have shown that *P. aeruginosa* infection results in a larger wound area, inhibiting healing, and a high prevalence of antimicrobial resistance. Hydroxypyridinone-derived iron chelator Deferiprone (Def) and heme analogue Gallium-Protoporphyrin (GaPP) in a chitosan-dextran hydrogel (Chitogel) have previously been demonstrated to be effective against PAO1 and clinical isolates of *P. aeruginosa* in vitro. Moreover, this combination of these two agents has been shown to improve sinus surgery outcomes by quickly reducing bleeding and preventing adhesions. In this study, the efficacy of Def-GaPP Chitogel was investigated in a *P. aeruginosa* biofilm-infected wound murine model over 6 days. Two concentrations of Def-GaPP Chitogel were investigated: Def-GaPP high dose (10 mM Def + 500 µg/mL GaPP) and Def-GaPP low dose (5 mM Def + 200 µg/mL GaPP). The high-dose Def-GaPP treatment reduced bacterial burden in vivo from day 2, without delaying wound closure. Additionally, Def-GaPP treatment decreased wound inflammation, as demonstrated by reduced neutrophil infiltration and increased anti-inflammatory M2 macrophage presence within the wound bed to drive wound healing progression. Def-GaPP Chitogel treatment shows promising potential in reducing *P. aeruginosa* cutaneous infection with positive effects observed in the progression of wound healing.

## 1. Introduction

*Pseudomonas aeruginosa,* a ubiquitous and opportunistic pathogen, is a Gram-negative bacterium known for its remarkable adaptability and resilience in diverse environments. *P. aeruginosa* is a common cause of wound infection and poses a substantial challenge in clinical settings due to its high resistance to antimicrobials and ability to form persistent and chronic infections resulting in high morbidity and mortality [1,2,3,4]. The intrinsic antimicrobial resistance of *P. aeruginosa* to multiple antibiotics makes this a difficult pathogen to treat and manage clinically [5,6]. Moreover, *P. aeruginosa* has been shown to utilise a number of mechanisms to continue to adapt to changing environmental conditions, producing biofilms that allow infection to persist and being able to adapt different iron acquisition methods based on iron availability in the microenvironment [4,7,8,9]. Iron is an important nutrient for *P. aeruginosa,* essential for various cellular functions, including respiration, DNA synthesis, protection against reactive oxygen species (ROS), and pathogenicity [10,11,12,13,14,15]. Additionally, intracellular iron acts as an important signal for *P. aeruginosa* biofilm development [16]. Eliminating bacterial biofilm is essential, as these infections continue to cause medical challenges with the increased antibiotic resistance [17].

Twitching motility in *P. aeruginosa* is also triggered in response to low iron availability, a mechanism designed to deter the bacterium from attaching to wound surfaces. This dynamic response effectively hinders the formation of microcolonies and subsequently prevents the development of biofilms [10,16]. Moreover, low iron uptake by *P. aeruginosa* during biofilm development can result in the formation of significantly thinner and more sparse biofilms compared with those grown with adequate iron availability [16]. *P. aeruginosa* employs various iron-uptake mechanisms, predominantly relying on siderophores that scavenge extracellular iron (Fe^3+^), binding extracellular iron and facilitating extracellular iron transport into the bacterial cell [16,18]. Further extracellular iron is also obtained from haem, which is acquired by the iron transport proteins present in the bacterial membrane and cleaved to release iron in the bacterial cell [19]. However, haem uptake has been shown to be low in *P. aeruginosa* when extracellular iron availability is high [20,21]. This interplay between iron requirements and haem uptake underscores the potential efficacy of a combined treatment strategy utilising iron chelator Deferiprone (Def) and haem analogue Gallium-Protoporphyrin (GaPP) to combat *P. aeruginosa* biofilm formation in cutaneous wounds. This approach capitalises on the intricate iron dynamics within *P. aeruginosa*, offering a promising avenue for disrupting biofilm development in clinical settings. Def reduces iron availability by forming a complex with free iron at a ratio of 3:1, with three Def molecules to one iron [22].

**Scheme 1:**
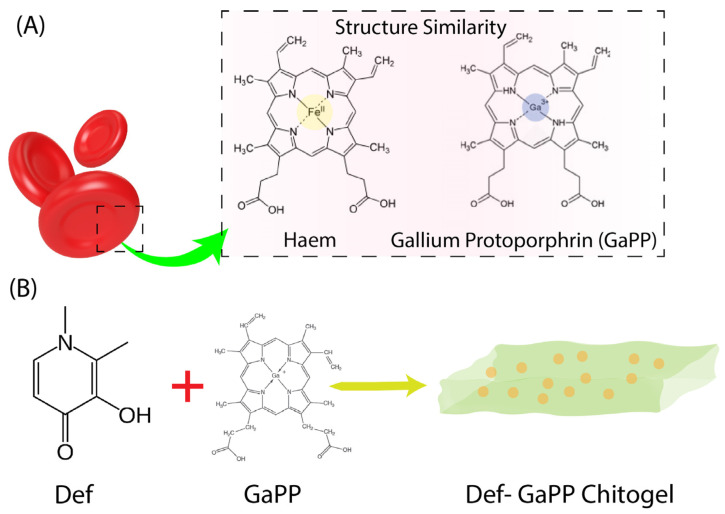
(**A**) Schematic illustration of haem and GaPP structural similarities. (**B**) The structure of Def combined with GaPP to be incorporated into Chitogel for wound healing applications.

The competition for iron between Def and *P. aeruginosa* therefore reduces extracellular iron availability and promotes the uptake of haem and consequently GaPP by the bacteria [20]. GaPP is a non-iron metalloporphyrin, which has a similar structure to haem, as shown in Scheme 1A [23]. However, GaPP cannot be cleaved by bacterial enzymes and this deficiency inhibits crucial bacterial functions, leading to impaired respiration, DNA synthesis, protection against reactive oxygen species (ROS), inhibited biofilm formation, reduced pathogenicity, and eventual cell death [10,11,12,13]. The Def and GaPP interaction offers a targeted strategy for disrupting essential bacterial processes, showcasing potential applications in inhibiting biofilm formation in clinical wound management.

The in vitro antibacterial potential of Deferiprone (Def) and Gallium-Protoporphyrin (GaPP) has been validated using both PAO1 and clinical isolates of *Pseudomonas aeruginosa* [21]. Notably, the sequential application of Def-GaPP treatment has exhibited superior antimicrobial effects compared with simultaneous application, underscoring the potential for enhanced therapeutic outcomes through a strategic and sequential administration of these agents [21]. However, studies to date have not investigated the translational potential of Def-GaPP Chitogel treatment combination using wound bacteria in a preclinical infection model of *P. aeruginosa* nor assessed subsequent wound healing outcomes. Previous studies have shown that the sequential application of Def and GaPP can be facilitated using biocompatible Chitogel, a highly effective dissolvable haemostatic hydrogel renowned for excellent release properties, high loading capacity, and advantageous wound-promoting characteristics, making this hydrogel a particularly well-suited delivery system for topical cutaneous wound applications (Scheme 1B). Chitogel exhibits a more rapid release of Def compared with GaPP, enabling a consecutive Def-GaPP treatment within a single hydrogel application [21]. Additionally, Def-GaPP Chitogel has been demonstrated to improve sinus surgery outcomes by quickly reducing bleeding and preventing adhesions, offering promising applications in both clinical wound care and surgical interventions [24].

The current study aimed to investigate the efficacy of Def-GaPP Chitogel treatment on the reduction of established chronic *P. aeruginosa* biofilm wound infection using both in vitro and in vivo assays, leveraging the bioluminescent strain of *P. aeruginosa* (Xen41). Our hypothesis of Def-GaPP Chitogel treatment decreasing wound infection and subsequent tissue inflammation was supported. In vivo antibacterial analysis showed that the Def-GaPP Chitogel has great potential to significantly decrease *P. aeruginosa* cutaneous wound infection compared with controls. Def-GaPP Chitogel treatment showed promise at reducing infection without delaying wound closure. Additionally, Def-GaPP treatment demonstrated reduced tissue inflammation in infected wounds, shown by reduced neutrophil infiltration and increased anti-inflammatory M2 macrophage phenotypes within the wound bed. Overall, the application of a high dose of Def-GaPP Chitogel (10 mM–500 µg/mL) holds considerable promise as a non-antibiotic strategy for addressing biofilm-associated cutaneous infections, offering a potential solution to the increasing complexities faced in global wound care.

## 2. Materials and Methods

### 2.1. Deferiprone-Gallium-Protoporphyrin Chitogel Preparation

Chitogel was prepared similarly to the previously described method [21,25]. Briefly, hydrogel treatments were prepared using a chitosan-dextran-based hydrogel (Chitogel). All the chemicals were commercially available and used without any further modifications or purification. Briefly, dextran aldehyde (DA) (300 mg) and succinyl chitosan (SC) in sodium phosphate buffer were combined with 0.3% NaPhos/40% glycerol buffer to form the Chitogel after overnight incubation at 4 °C. When Def (Sigma, Castle Hill, Australia) or GaPP (Frontier Scientific, Logan, UT, USA) was loaded into the Chitogel, 5 mL of the sodium phosphate buffer was replaced with 5 mL Def or GaPP at four times the desired concentration. For example, to make Def 10 mM Chitogel, Def 40 mM was added. When both Def and GaPP were added to the Chitogel, dextran aldehyde was dissolved in Def (5 mL) and GaPP (5 mL). To the dissolved dextran aldehyde, 5% succinyl chitosan in sodium phosphate buffer (10 mL) was added and quickly combined. The combined mixture was poured into a petri dish lid (9 cm diameter) and then covered with aluminium foil to protect from light and allowed to set overnight at room temperature. Four Chitogel preparations were used for in vitro antimicrobial testing: Chitogel (vehicle control), 10 mM Def Chitogel, 500 µg/mL GaPP Chitogel, and 10 mM Def + 500 µg/mL GaPP Chitogel. Two Def-GaPP Chitogel preparations were prepared for in vivo antimicrobial and wound healing testing: 5 mM Def + 200 µg/mL GaPP and 10 mM Def + 500 µg/mL GaPP.

### 2.2. Deferiprone-Gallium-Protoporphyrin Chitogel Characterisation

The Def-GaPP Chitogel was characterized similarly to our previous work [26]. The pH was measured using pH strips dipped into the Chitogel mixture after combining all components. Rheological properties of the Chitogel and Def-GaPP Chitogel were assessed using a rheometer (TA Instrument, New Castle, DE, USA) and a 25 mm diameter parallel plate. An oscillatory shear rate sweep was conducted at 25 °C and a shear rate of 1–100 s^−1^. The microstructure of Chitogel was examined using Hitachi Tabletop scanning electron microscopy (SEM, Hitachi Tabletop SEM TM4000Plus, Tokyo, Japan). The samples were freeze-dried and mounted on SEM stubs and imaged at different magnifications. 

### 2.3. Minimum Inhibitory Concentration

The Def-GaPP Chitogels were subjected to antibacterial tests specifically targeting a selected Gram-negative pathogen, *P. aeruginosa* Xen41, a bioluminescent strain highly relevant to cutaneous wound infections. Minimum inhibitory concentration (MIC) of Def and GaPP liquid treatments against *P. aeruginosa* (Xen41) were determined by the micro-broth dilution method, similar to previously published protocols [25]. Treatment concentrations started at Def: 20 mM, GaPP: 1000 µg/mL, and Def-GaPP: 10 mM–500 µg/mL. The MIC was determined as the minimum concentration to prevent bacterial growth over 24 h.

### 2.4. Zone of Inhibition 

The zone of inhibition of Def and GaPP Chitogel treatments was determined by using a disk diffusion assay, as previously described [26]. Briefly, 100 µL 1 × 10^5^ CFU/mL *P. aeruginosa* was spread on the surface of tryptic soy agar (TSA) plates. Treatments were applied to the agar plate Chitogel, Def Chitogel: 10 mM, GaPP Chitogel: 500 µg/mL, and Def-GaPP Chitogel: 10 mM–500 µg/mL. The zone of inhibition was measured after 24 h incubation at 37 °C.

### 2.5. In Vitro Wound Biofilm Assay

The in vitro wound biofilm assay previously described [26,27] was used to investigate the antimicrobial effects of Def and GaPP on mature established *P. aeruginosa* biofilms. Briefly, a biofilm was grown on a polycarbonate membrane on brain-heart-infusion agar with artificial wound fluid (fetal calf serum (FCS) with 1% peptone water). After incubation at 37 °C for 24 h, Def and GaPP Chitogels (Chitogel, Def Chitogel: 10 mM, GaPP Chitogel: 500 µg/mL, and Def-GaPP Chitogel: 10 mM–500 µg/mL) were applied to the mature biofilms, and untreated biofilms were used as the control. The biofilms were incubated with treatments for a further 24 h, and the bacteria were then suspended in PBS plated on TSA and incubated overnight at 37 °C. Colony counts were conducted to determine the colony-forming units (CFU) present in the biofilms.

### 2.6. Crystal Violet Biofilm Quantification 

The effect of Def and GaPP Chitogel on initial bacterial attachment was investigated by crystal violet assay following established protocols [26]. *P. aeruginosa* bacterial culture was adjusted with PBS and 1 mL of 1.0 × 10^5^ CFU/mL was added to 12 well plates containing Transwell inserts (Corning^®^ Transwell 0.4 µm pore polyester membrane inserts, Corning, NY, USA). Def and GaPP Chitogel treatments (Chitogel, Def Chitogel: 10 mM, GaPP Chitogel: 500 µg/mL, and Def-GaPP Chitogel: 10 mM–500 µg/mL) were added to the Transwell at air-liquid interface and the plates were incubated at 37 °C. After 24 h, the wells were gently washed and stained with crystal violet (0.1%). The crystal violet-bound cells were solubilised with 30% methanol and 10% acetic acid solution, and the released stain was measured at 550 nm using a microplate reader (ELx800 Microplate Reader, BioTek, Santa Clara, CA, USA) to quantify bacterial biofilm attachment [28]. 

### 2.7. In Vitro Cytotoxicity and Wound-Healing Effects of Treatments

The effect of Chitogel, Def Chitogel: 10 mM, GaPP Chitogel: 500 µg/mL, and Def-GaPP Chitogel: 10 mM–500 µg/mL on human skin cell proliferation, scratch-wound migration, and cytotoxicity was assessed using both human foreskin fibroblasts (HFFs; HFF-1, ATCC^®^ SCRC1041™, Manassas, VA, USA) and human keratinocytes (HaCaTs; CLS Cell Lines Service, 300493, Eppelheim, Germany). Cells were grown in Dulbecco’s Modified Eagle’s Medium (DMEM; Life Technologies, Carlsbad, CA, USA) with 10% FCS and 5% penicillin and streptomycin and incubated at 37 °C in 5% CO_2_.

#### 2.7.1. MTT Assay

The effect of treatments on skin cell proliferation was determined using an MTT assay. Cells were seeded at 5 × 10^4^ cells/well in a 12-well Transwell plate and incubated for 24 h, following which cell cycles were synchronised by replacing the cell culture medium with FCS-free DMEM for 6 h. After synchronisation, the cell culture medium, including 10% FCS and the above-mentioned treatments, was added to wells and incubated for 24 h. Following incubation, the treatments and media were removed and 1 mL MTT (0.5 µg/mL) was added to each well for a 2 h incubation, then removed, and Dimethyl sulfoxide (DMSO) was added. After 15 min the absorbance was read at 570 nm on a FLUOstar OPTIMA plate reader (BMG LabTech, Melbourne, Australia). Percent proliferation relative to the control was calculated.

#### 2.7.2. Scratch-Wound Assay

The effect of treatments on human skin cell migration was determined using a standard scratch-wound assay. Cells were seeded at 2 × 10^5^ cells/well in a 12-well plate and incubated at 37 °C overnight. Once the cells were confluent, wounds were created on the cell monolayers using an Incucyte WoundMaker (Sartorius, Germany). Then, the cells were washed with PBS before adding treatments and DMEM with 10% FCS. Cells were incubated and imaged every 3 h using the Olympus IX83 Fluorescence Microscope (Olympus, Tokyo, Japan) for 12 h and again at 24 h and 30 h. The distance between cell fronts was measured using the ImageProPlus program (Media Cybernetics Inc., Bethesda, MD, USA) and percentage wound closure was calculated.

#### 2.7.3. Resazurin Cytotoxicity Assay

The cytotoxicity of Def and GaPP Chitogels on human skin cells was tested using a resazurin assay [26]. Cells were seeded 2 × 10^5^ cells/well in 12-well Transwell inserts and incubated for 24 h. The cells were then washed with PBS, treatments were added, and the cells were incubated for a further 24 h. The cells were washed again with PBS, and then 1 mL of 10% resazurin solution (stock 110 µg/mL) was added and the cells incubated for 2 h. The absorbance of fluorescence intensity was measured in each well at an excitation wavelength of 540 nm and emission spectra of 590 nm, on a FLUOstar OPTIMA plate reader (BMG LabTech, Melbourne, Australia). Percent cell viability relative to the control was calculated and cell viability less than 70% was considered cytotoxic. Resazurin solution alone acted as a negative control for background corrections.

### 2.8. Animal Ethics

All animal experiments were approved by the University of Adelaide’s animal ethics committee (Animal Ethics Number: M-2018-081). The study was completed in compliance with the Australian Code of Practice for the Care and Use of Animals for Scientific Purposes (8th Edition 2013) and the South Australian Animal Welfare Act 1985.

### 2.9. In Vivo Antimicrobial Efficacy of Def-GaPP Chitogel

The in vivo antimicrobial treatment efficacy of Def-GaPP Chitogel treatments on established *P. aeruginosa* (Xen41) wound biofilms was investigated using a preclinical wound infection murine model [29]. Male and female Balb/c mice obtained from the ARC at 12 weeks of age were separated into 5 treatment groups (*n* = 8/group): non-infected control, infected control, antibiotic control (Ciprofloxacin 0.3% 3 mg/g), Def-GaPP low (5 mM–200 µg/mL), and Def-GaPP high (10 mM–500 µg/mL). On day 0 of the study, all mice were anaesthetised using 2% isoflurane, and excisional wounds were created at the centre of the back using a 6 mm punch biopsy. All wounds, excluding the non-infected control group, were inoculated with 1 × 10^4^ CFU of *P. aeruginosa* (Xen41). Digital images of wounds and bacterial infection were captured using the Xenogen IVIS Bioluminescent Live Animal Imaging System (Caliper Life Sciences, Hopkinton, MA, USA) immediately following inoculation. Following imaging, the wounds were covered with a Tegaderm dressing to facilitate biofilm growth. Following day 1, once biofilms were established, all mice underwent daily treatment, imaging, and reapplication of dressings until the conclusion of the study. Non-infected controls and infected controls received no treatment. On day 6 of the study following imaging, all mice were humanely killed via cervical dislocation, and wounds were collected for histology, quantification of CFU, and biofilm biomass following established protocols [30].

### 2.10. CFU Quantification

Wound tissue collected on day 6 was homogenised in PBS through a series of sonication and vortex steps to isolate wound bacteria into the solution. The homogenised solution was then 10-fold serial diluted and plated on selective Luria-Bertani agar plates with 10 µg/mL Tetracycline. Plates were incubated overnight at 37 °C and imaged using Xenogen IVIS Bioluminescent Live Animal Imaging System (Caliper Life Sciences, Hopkinton, MA, USA). Bacterial colonies were counted, and CFU/g tissue was calculated following established protocols [30].

### 2.11. Biofilm Biomass Analysis

LIVE/DEAD biofilm staining was performed on collected wounds to assess biofilm biomass following established protocols [30]. Wound tissue collected on day 6 was kept in sterile DMEM on ice prior to analysis. The sections were washed and stained using a LIVE/DEAD BacLight Bacterial Viability Kit (Life Technologies Australia, Victoria, Australia) according to the manufacturer’s manual. Stained tissue sections were imaged at 20× magnification using a confocal scanning laser microscope (Zeiss LSM700, Carl Zeiss AG, Oberkochen, Germany). Three Z-stack images (40 slices, interval 2) were then taken from each wound tissue section. COMSTAT version 2.1 (www.comstat.dk accessed on 28 September 2022; [31,32]) was used to measure the biofilm biomass, with the threshold set manually to minimise background staining following established protocols [24].

### 2.12. In Vivo Wound Promoting Efficacy of Def-GaPP Chitogel

Assessment of Def-GaPP Chitogel treatment efficacy on wound healing was determined by both macroscopic and histological analysis of wound healing, as described previously [30]. Wound samples were fixed in 10% neutral buffered formalin, embedded in paraffin wax, and cut at 4 µm thickness. Sections were then hematoxylin and eosin (H&E) stained, and wound length, dermal gape and re-epithelialisation were measured using the ImageProPlus program (Media Cybernetics Inc., Bethesda, MD, USA). Additional cut wound sections were processed for immunohistochemistry using previously established methods [30]. Sections were then deparaffinised and underwent antigen retrieval using target retrieval solution (TRS) in an Antigen Decloaker (Biocare Medical, Pacheco, CA, USA) at 90 °C for 10 min. Next, sections were blocked in 3% normal goat serum (NGS) for 30 min. Primary antibodies (Table S1) were then applied in 3% NGS in PBS overnight at 4 °C. After incubation, secondary antibodies (Table S2) were applied for 1 h at room temperature, then nuclei stained with DAPI (Sigma Aldrich, Sydney, Australia) at a 1:5000 (1 mg/mL stock). All sections were imaged using an Olympus IX83 Fluorescence Microscope (Olympus, Tokyo, Japan). Quantification of immunohistochemical staining included assessment of total positive cell numbers within the wound bed using the ImageProPlus 7.0 program. 

### 2.13. Statistical Analysis

Results are presented as mean ± standard deviation (SD) unless otherwise stated. Data were analysed by Student’s t-test or one-way ANOVA. When the statistical analysis was significant (*p* < 0.05), post hoc comparisons were conducted using Dunnett’s multiple comparisons. All statistical analysis was performed using GraphPad Prism version 8.0 (GraphPad, Boston, MA, USA). * p < 0.05, ** p < 0.01 and *** p < 0.001.

## 3. Results and Discussion

### 3.1. Characterisations of Def-GaPP Chitogel 

**Figure 1:**
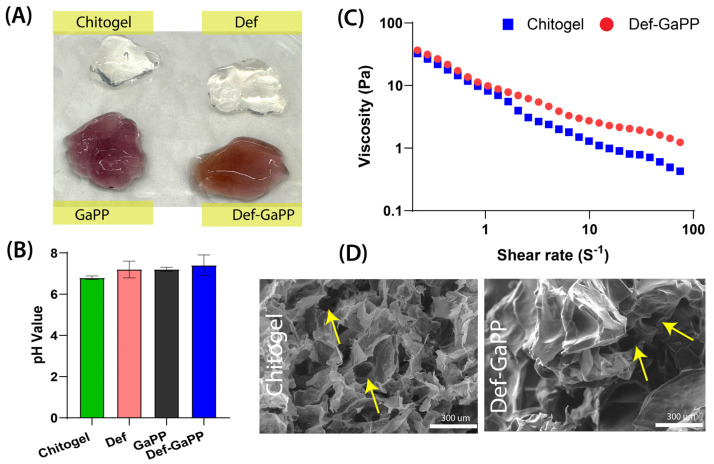
Characterisations of the Chitogel and Def-GaPP Chitogel. (**A**) Photograph of the final formulations. (**B**) The pH measurements of each formulation. (**C**) The shear rate against viscosity of the final Chitogel and Def-GaPP Chitogel. (**D**) The microstructure of the Chitogel and Def-GaPP Chitogel analysed by SEM, Yellow arrows indicate pores in the Chitogel structure, scale 300 μm.

The Chitogel was prepared using previously established methods [21,25]. The final formulation was obtained by mixing an optimized ratio of dextran aldehyde (DA) (300 mg) and succinyl chitosan (SC) in sodium phosphate buffer combined with 0.3% NaPhos/40% glycerol buffer to form the Chitogel. The medicated Chitogel was prepared by adding either Def, GaPP, or combined Def-GaPP, as described in the methods section. The final formulation was consistent, soft, and pliable, with ease of application for wound purposes, as shown in (Figure 1A). The pH of the formulation was tested using a commercial pH strip with no major difference between the groups, except for the Chitogel blank having a slightly lower pH of ~7 (Figure 1B). The rheology of the Chitogel and Def-GaPP was assessed using a rheometer following established protocols [26]. As shown in Figure 1C, as the shear rate increased, the viscosity of the gels reduced, with no major difference between the Chitogel or Def-GaPP Chitogel groups. This is expected with this type of hydrogel. The interior microstructure of the hydrogel was also assessed using SEM. As shown in Figure 1D, the Chitogel is highly porous with visible pores (yellow arrow) and a honeycomb-like structure similar to the Def-GaPP Chitogel, suggesting no differences in the crosslinking degree of gel matrices. 

### 3.2. In Vitro Inhibition of P. aeruginosa Growth by Def-GaPP Chitogel Combination

The application of Deferiprone (Def) and Gallium-Protoporphyrin (GaPP) in antibacterial treatments presents a promising avenue, and we validated its safety and efficacy for addressing *P. aeruginosa* cutaneous infections of established wound biofilms. Previous studies have demonstrated that the combination of Def and GaPP effectively inhibits bacterial growth with increased efficacy when applied in combination rather than as individual treatment [21]. The MIC of Def and GaPP liquid treatments against *P. aeruginosa* (Xen41) in the current study was determined by the micro broth dilution method. Consistent with our previous studies, the MICs of Def and GaPP against *P. aeruginosa* in the current study were lower when used in combination compared with individual treatments. The MIC of individual treatments is presented in Table S3, showing that combined treatment had a much lower MIC of Def-GaPP 2.5 mM–125 µg/mL. The combination treatment resulted in a 4-fold reduction of MIC, indicative of a synergistic property as determined by fractional inhibitory concentration analysis (Table S3). This improved antimicrobial efficacy of combined treatment compared with individual treatment was also observed in the zone of inhibition assays, which showed a significantly larger zone of clearance when treated with Def-GaPP Chitogel. Quantitative analysis showed that Def Chitogel and Def-GaPP Chitogel treatments resulted in a 10.8 mm and 20.3 mm zone of inhibition, respectively. However, Chitogel and GaPP Chitogel treatment did not inhibit *P. aeruginosa* growth after 24 h (Table S4). These in vitro results are consistent with previous studies showing combined Def-GaPP Chitogel treatment to have better antimicrobial properties than individual treatments, and that sequestering of extracellular iron by Def is a critical mechanism underlying these results [19,21]. Moreover, these results indicated that combined treatment applied in a Chitogel delivery system was optimal for inhibition of *P. aeruginosa* growth.

### 3.3. Def-GaPP Chitogel Reduces P. aeruginosa Bacterial Biofilms In Vitro

**Figure 2:**
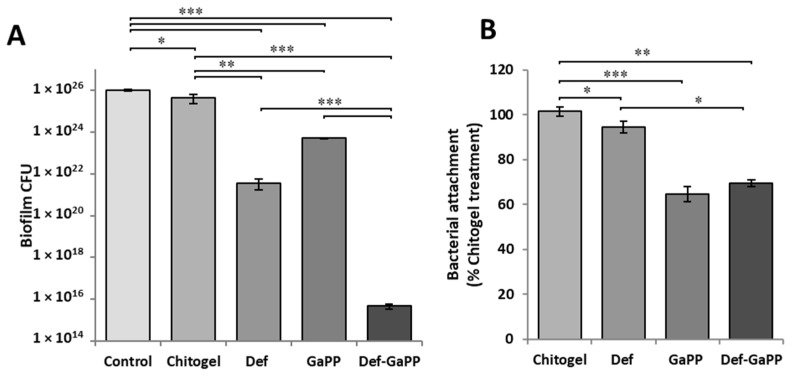
In vitro antibacterial analysis of the Chitogel, Def, GaPP and Def-GaPP treatment groups. (**A**) Quantification of *P. aeruginosa* biofilms CFU 24 h post-treatment with Def and GaPP Chitogels in a wound biofilm model. (**B**) *P. aeruginosa* biofilm attachment measured via crystal violet assay in response to treatment after 24 h compared with controls. Data are shown as mean ± SEM. * *p* < 0.05, ** *p* < 0.01 *** *p* < 0.001.

The in vitro anti-biofilm effect of the Def-GaPP Chitogel treatments against *P. aeruginosa* was also assessed using a biofilm assay [26]. The biofilms were quantified using CFU counts. All treatments showed significantly reduced bacterial CFUs, with the largest reduction observed in the combined Def-GaPP Chitogel-treated biofilms (Figure 2A). Both individual and combined Def and GaPP treatments slightly reduced bacterial CFUs compared with the Chitogel control treatment. Therefore, the combined Def-GaPP treatment was highly effective in significantly reducing *P. aeruginosa* biofilms, suggesting its potential as a strategic approach for combatting biofilm-associated challenges. This observation was further verified using a bacterial attachment assay showing individual and combined Def and GaPP treatments to reduce *P. aeruginosa* attachment compared with Chitogel control treatment. The combined Def-GaPP treatment significantly reduced bacterial attachment compared with control treatments (Figure 2B). This finding agrees with previous studies that have shown that *P. aeruginosa* requires iron for bacterial attachment [20,33]. Despite most of the iron acquired by *P. aeruginosa* being scavenged by siderophores, haem is also used when iron availability is lower than required by the bacteria [16,18]. Bacterial attachment is the first step toward biofilm development, which requires iron [16]. Increased iron requirement for biofilm development may have therefore resulted in an increased uptake of GaPP by *P. aeruginosa*, resulting in a similar bacterial attachment between GaPP and Def-GaPP treatments. These in vitro anti-biofilm observations indicated that a combination of Def-GaPP Chitogel could be used as an anti-biofilm treatment strategy to eradicate biofilms from a colonised wound surface. 

### 3.4. Def-GaPP Chitogel Is Safe to Use on Human Skin Cells In Vitro 

**Figure 3:**
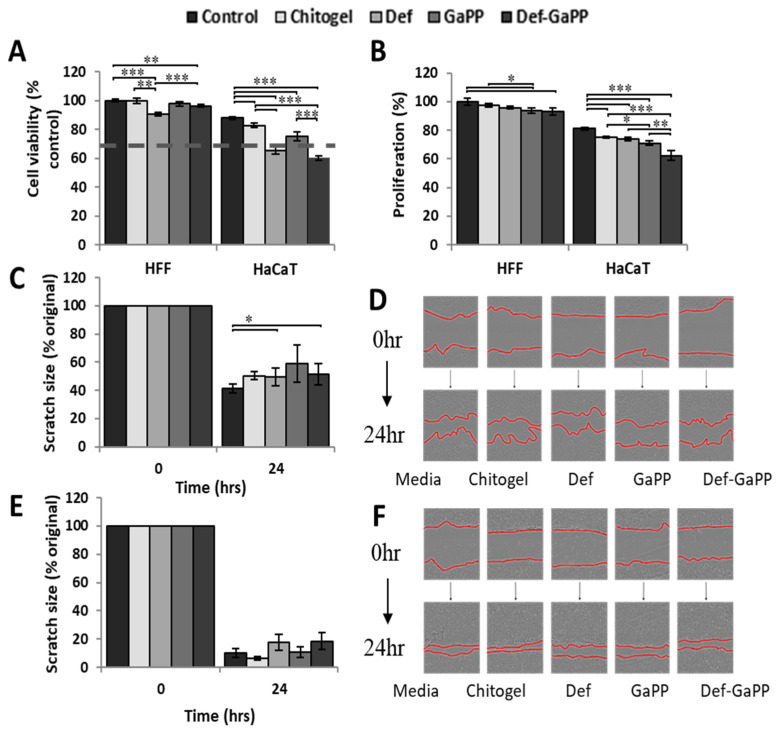
In vitro analysis of cell viability, proliferation, and migration in response to treatment with Def and GaPP Chitogels after 24 h. (**A**) Fibroblast and keratinocyte cell viability after 24 h treatment. (**B**) Fibroblast and keratinocyte cell proliferation. (**C**,**D**) The cell migration capacity of fibroblast at different time points with representative images. (**E**,**F**) The cell migration capacity of keratinocytes at different time points with representative images. The dashed line represents the industry standard cut-off for skin cell viability against biomaterials; viability above 70% is considered clinically safe for use. Data shown as mean (%) ± SEM. * *p* < 0.05, ** *p* < 0.01 *** *p* < 0.001.

Def and GaPP Chitogel biocompatibility was tested in vitro on fibroblasts (HFF) and keratinocytes (HaCaT) by measuring the cell metabolic activity in a Resazurin assay following the ISO 10993-5 standard [34] for indirect in vitro cytotoxicity testing [26]. As shown in Figure 3A, compared with controls, the HFFs remained over 95% viable when treated individually or with combined treatment. A similar result was observed in response to HaCaTs, except there was a slight reduction of viability against the combined Def-GaPP treatment (Figure 3A). However, the cell viability was approximately 70%, suggesting a biocompatible nature following the international ISO 10993-5 standard of biomaterial testing for in vitro cytotoxicity. Additionally, these results support previous in vitro studies that demonstrated that liquid Def treatment showed no cytotoxicity up to 10 mM on primary human nasal epithelial cells and human nasal fibroblasts [35], and up to 20 mM in mouse fibroblasts (L929) and human airway epithelial (Nuli-1) cells [19]. Moreover, a previous study has shown GaPP to have no cytotoxicity below 500 µg/mL on L929 and Nuli-1 cells [19], also supporting the use of selected concentrations for both agents in the current study.

Biocompatibility was further investigated in vitro by proliferation and scratch-wound migration assays using skin cells. Proliferation was investigated by MTT proliferation assay and demonstrated similar results to the resazurin cell viability assay. There was a minimal reduction of fibroblast proliferation from all treatments, with the largest proliferation reduction compared with the untreated control being a 5% reduction in Def-GaPP-treated cells (Figure 3B). Interestingly, consistent with the effects on cell viability, keratinocyte proliferation showed a larger reduction compared with the untreated control. In these cells, the greatest proliferation reduction compared with the untreated control was 25% in Def-GaPP-treated cells (Figure 3B). However, despite reduced proliferation, the scratch-wound assay showed that treatments had a minimal effect on cell migration. Fibroblast migration was reduced by Def and Def-GaPP (Figure 3C,D), which is consistent with a previous study that showed Def slowed the migration of primary human nasal epithelial cells and human nasal fibroblasts [35]. Importantly, none of the treatments slowed the migration of keratinocytes after 24 h, showing no effects on the rate of wound closure, which is critical for effective wound re-epithelisation (Figure 3E,F). The analysis of cell morphology and rate of scratch-wound closure showed no effects on cell function or morphology suggestive of healthy growing cells (Figure 3D,F). These results suggest that the combination of Def-GaPP Chitogel at the concentration used in this study could be applied as a safe therapeutic approach for treatment of cutaneous bacterial infections without impairing cellular responses vital for tissue regeneration.

### 3.5. High Dose Def-GaPP Chitogel Reduces the Bacterial Burden of P. aeruginosa In Vivo

**Figure 4:**
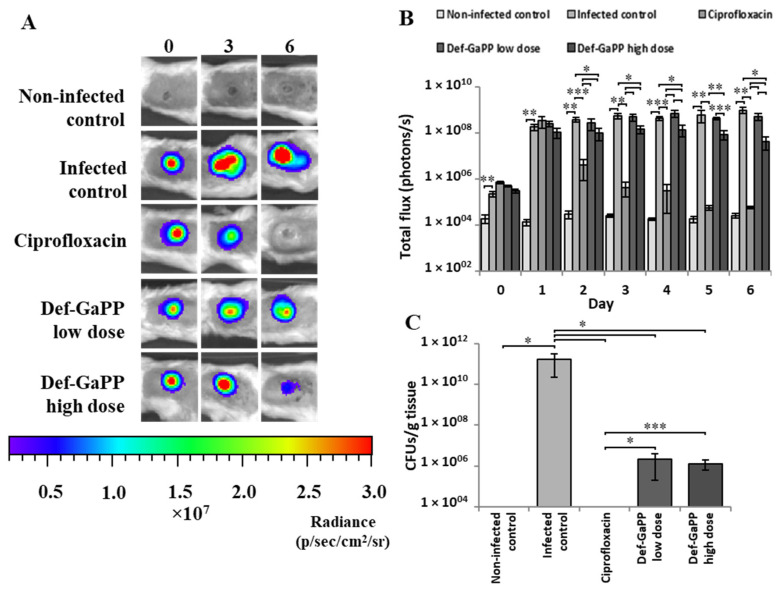
In vivo analysis of the *P. aeruginosa* (Xen41) biofilm infection wound model following treatment with Ciprofloxacin, Def-GaPP (5 mM–200 µg/mL) low dose, and Def-GaPP (10 mM–500 µg/mL) high dose over 6 days. (**A**) A representative bioluminescent image showing the progression of infection in response to treatment. (**B**) Quantification of bacterial burden expressed as the average total flux (photons/s). (**C**) Bacterial count from the wound tissue collected after the endpoint (day 6). Data shown as mean ± SEM. * *p* < 0.05, ** *p* < 0.01, *** *p* < 0.001, *n* = 8.

The in vivo antibacterial effect of Def, GaPP, Chitogel, and Def-GaPP Chitogel treatment was investigated using the *P. aeruginosa* wound biofilm infection model, with treatment over 6 days. Quantification of bacterial burden (metabolically active) bacteria was carried out using the IVIS live imaging system daily (Figure 4A) [30]. Quantitative analysis showed that the bacterial burden of mice treated with the antibiotic ciprofloxacin cleared infection within 5 days (Figure 4B). These wounds had a low total flux similar to the non-infected control mice that showed no infection, consistent with our previous findings in the *S. aureus* biofilm wound infection model (Figure 4B) [30]. Ciprofloxacin is a broad-spectrum antibiotic that is used clinically to treat *P. aeruginosa* infections because of the antibiotic’s ability to penetrate *P. aeruginosa* biofilms with a higher inhibition rate efficiency [36,37]. This antibiotic is commonly used to treat urinary tract infections and pneumonia. The eradication of the *P. aeruginosa* infection within 5 days indicates that the employed Ciprofloxacin dosage is clinically relevant for combatting bacterial infections within a short duration. Nevertheless, prolonged use of Ciprofloxacin could potentially elevate the risk of bacterial resistance, as bacteria, especially *P. aeruginosa*, are known to develop resistance over time with repeated applications [38]. However, Def-GaPP exhibited a dose-dependent reduction in bacterial burden compared with the infected control. Specifically, the high dose of Def-GaPP significantly decreased bacterial burden from day 2 onward compared with the infected control, whereas the low-dose treatment required a longer duration to manifest a similar effect in vivo and the number of metabolically active bacteria was significantly lower in high-dose-treated groups on experimental days 4–6 compared with control (Figure 4B). The influence of sex on the results of the study was not observed between male and female mice. On day 6 of the experimental period, the antimicrobial effect of high-dose Def-GaPP on bacterial burden was further confirmed using CFU counts on collected wound tissue to quantify the total number of *P. aeruginosa* bacteria present (metabolically active and planktonic bacteria in wound biofilms) (Figure 4C). A similar trend was observed in reducing bacterial counts, with no significant difference between the two Def-GaPP doses tested, while antibiotic treatment significantly reduced bacterial numbers (Figure 4C).

**Figure 5:**
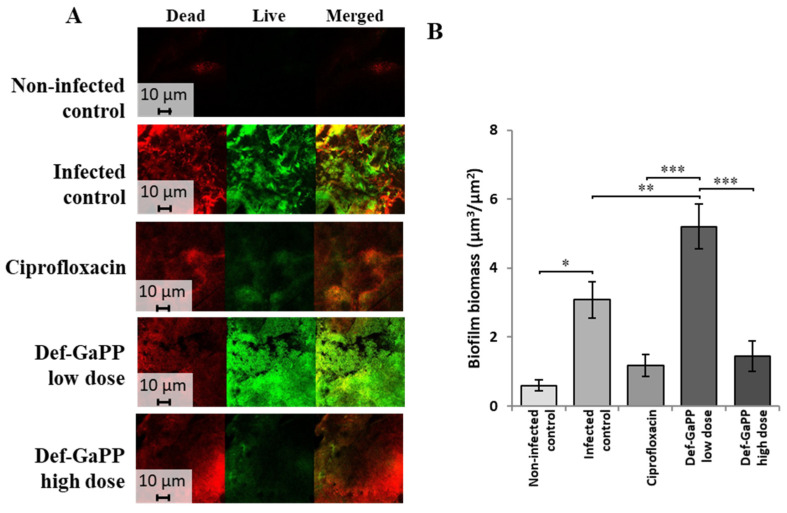
In vivo biofilm biomass analysis of *P. aeruginosa* infection after 6-day treatment with Ciprofloxacin, Def-GaPP (5 mM–200 µg/mL) low dose, and Def-GaPP (10 mM–500 µg/mL) high dose. (**A**) Representative confocal laser scanning microscope images of live (green) and dead (red) bacteria in *P. aeruginosa* infected wounds. (**B**) Corresponding Bacterial biomass (µm^3^/µm^2^). Data shown as mean ± SEM. * *p* < 0.05, ** *p* < 0.01, *** *p* < 0.001. Scale bar 10 µm, *n* = 8.

The bacterial biofilm collected from the wounds on the last day of the experiment was assessed using LIVE/DEAD Baclight^TM^ stain (Figure 5A). The CLSM images show the distribution of mass of biofilms in response to all treatment groups. The green fluorescence indicates the presence of viable bacterial biofilms, while red is indicative of dead bacteria. The biofilm biomass was quantified following established protocols [24]. As shown in Figure 5B, Ciprofloxacin-treated wound biofilm biomass was significantly reduced compared with the untreated infected control and was not significantly different from the non-infected control, as predicted, suggesting a high inhibition rate (Figure 5B). The effect of Def-GaPP treatment on biofilm biomass was observed to be dose dependent. Wounds treated with low-dose Def-GaPP had significantly increased biofilm biomass compared with the infected control, antibiotic control, and Def-GaPP high-dose groups. A high-dose treatment of Def-GaPP resulted in significantly lower biofilm biomass compared with the infected control, and importantly no difference in the antibiotic control treatment (Figure 5B). This analysis further supports the strong efficacy of the combined Def-GaPP treatment to target bacterial biofilms and significantly reduce bacterial viability and biomass at the optimised concentration.

### 3.6. Concentration-Dependent Treatment Effects on Wound Healing Progression 

**Figure 6:**
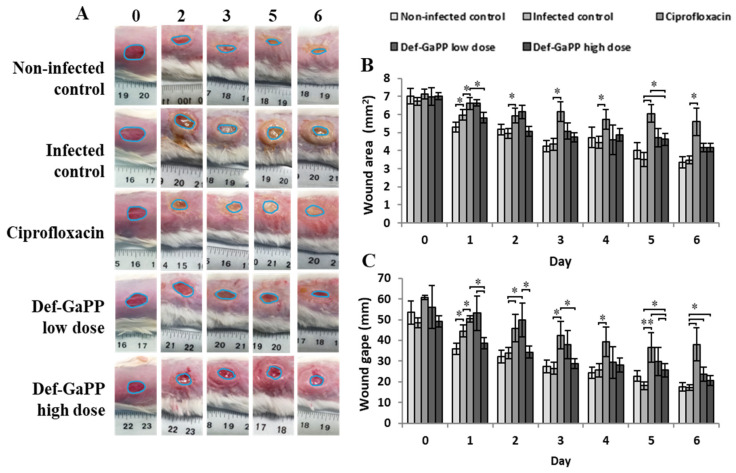
Analysis of wound healing in *P. aeruginosa*-infected wounds over 6 days of Ciprofloxacin, Def-GaPP (5 mM–200 µg/mL) low-dose treatment, and Def-GaPP (10 mM–500 µg/mL) high-dose treatment. (**A**) A representative macroscopic digital image of the wound site; the blue line represents wound area. (**B**) Wound area (mm^2^) over time. (**C**) Wound gape (mm) over time. Data shown as mean ± SEM. * *p* < 0.05, ** *p* < 0.01, *n* = 8.

Previous studies have shown that clinical wound infection delays healing by triggering an exaggerated inflammatory response, interfering with cellular activities, causing tissue damage, and promoting the formation of bacterial biofilms [39,40]. Addressing and managing clinical infections are crucial steps in promoting timely and effective wound healing [8]. Clearing infection safely and timely is crucial to allow normal healing to commence [30,41]. However, many antimicrobials have been shown to impair healing despite having strong antimicrobial properties that eliminate infection [42]. In this study, the effect of Def-GaPP Chitogel on *P. aeruginosa* cutaneous wound healing was analysed both macroscopically and histologically. Firstly, the macroscopic wound healing progression was investigated by analysis of wound area and gape (Figure 6). Representative images of the infected wounds show a raised area of maceration around the infected control wounds and the Def-GaPP treated wounds indicative of an established biofilm formation (Figure 6A). However, in the wounds treated with Def-GaPP the raised area had a more natural pink colour and was dry compared with the infected control wound where evidence of pus formation was observed, indicating that the Def-GaPP wounds were responding to treatment. Day 2 analysis showed larger wound areas and gape in the infected control compared with the non-infected control for this biofilm wound infection model however surprisingly this difference was not observed on other days of the trial. Ciprofloxacin antibiotic control treatment significantly delayed wound closure; these wounds had a significantly larger wound area and wound gape compared with the infected control from day 1 onward with the difference being more pronounced from day 3 until the end of the experiment (Figure 6B,C). This finding is in agreement with the literature regarding the negative effects of topical antibiotics on wound healing [43]. While wounds treated with Def-GaPP high dose showed a faster rate of reduction in wound gape in earlier time points this effect was not evident by the end of the study. Importantly, wounds treated with Def-GaPP at low and high doses overall showed a very similar wound-healing progression throughout the study (Figure 6A–C).

**Figure 7:**
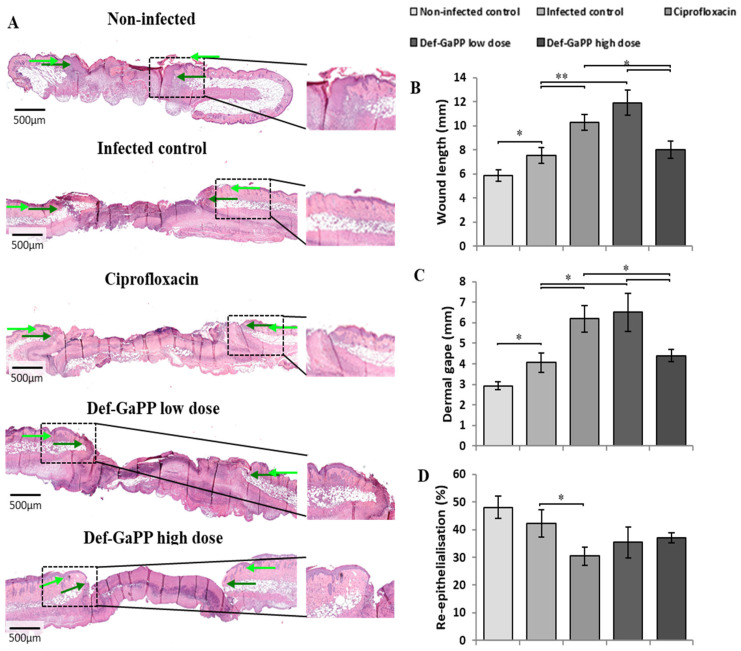
Histological analysis of wound healing in *P. aeruginosa*-infected wounds after 6 days of Ciprofloxacin, Def-GaPP (5 mM–200 µg/mL) low-dose treatment, and Def-GaPP (10 mM–500 µg/mL) high-dose treatment. (**A**) A representative image of histological wound sections; the dark green arrows indicate dermal gape, light green arrows represent wound length, and zoomed inserts show epithelial thickness. Analysis of (**B**) wound length, (**C**) dermal gape, and (**D**) re-epithelialisation. Data shown as mean ± SEM. * *p* < 0.05, ** *p* < 0.01. Scale bar 500 µm, *n* = 8.

Histological analysis of wound healing was used for observing granulation tissue formation and assessing key features of wound healing, including wound re-epithelisation. (Figure 7A). Representative wound sections show the wound microstructure, where the green arrow indicates wound length (light green) and dermal gape (dark green). The results indicated that the rate of wound healing is consistent with the macroscopic data. The wound length and dermal gape for antibiotic and low-dose Def-GaPP treatments were significantly higher compared with other treatments, including high-dose Def-GaPP treatment. Additionally, high-dose Def-GaPP treatment showed no significant difference in wound length or dermal gape compared with infected and non-infected control groups (Figure 7B,C). Wound re-epithelialisation was not significantly affected by *P. aeruginosa* infection, however, ciprofloxacin treatment significantly reduced the rate of wound re-epithelialisation compared with the infected control (Figure 7D). The high-dose Def-GaPP treatment reduced infection and did not significantly impair or improve healing compared with the infected control, suggesting this dose is highly antimicrobial and safe for use against *P. aeruginosa*-infected wounds. Overall, this study suggests that, to achieve optimal healing, the combination of Def-GaPP at the optimised concentration is vital to support the wound healing phases without being non-toxic. Wound healing is impacted by numerous factors, including the duration of the healing [44]. This study focused on a relatively short duration of treatment, specifically 6 days, to assess the healing effect of the proposed treatment. However, it is important to acknowledge that, in the clinical setting, infected wounds often require more than 10 days to complete the healing process. The results imply that extending the study duration could yield even more favourable outcomes, potentially leading to accelerated closure of wounds with enhanced tissue regenerative capacity.

### 3.7. Def-GaPP Chitogel Has No Effect on Keratinocyte Proliferation and Reduces Inflammation of P. aeruginosa-Infected Wounds

**Figure 8:**
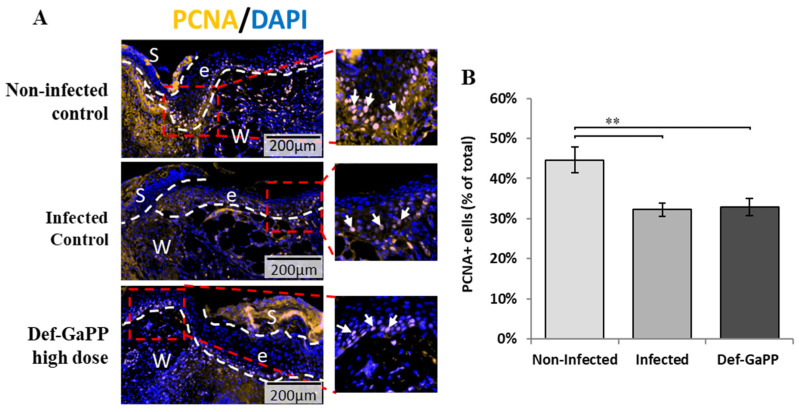
Analysis of proliferating cell nuclear antigen (PCNA)-positive epidermal keratinocytes on day 6 in a wound treated with Def-GaPP Chitogel. (**A**) Representative fluorescence images of PCNA-positive cells (PCNA (orange)/DAPI (blue)). (**B**) Corresponding analysis of PCNA-positive cells expressed as a percentage of total cell counts. The white dotted line defines the area of the neoepidermis, and white arrows indicate PCNA-positive cells; (s) scab, (e) epidermis, and (w) wound bed. Data shown as mean ± SEM. Scale bar 200 μm, ** *p* < 0.01, *n* = 8.

To further investigate the effects of high-dose Def-GaPP Chitogel on wound healing in a *P. aeruginosa* biofilm wound infection model, the proliferation of keratinocytes in the neoepidermis and analysis of inflammatory cells within the wound bed was undertaken. The wound sections were stained for specific markers in response to high-dose Def-GaPP alongside infected and non-infected controls. Sections stained for proliferating cell nuclear antigen (PCNA), showed that PCNA-positive cells in the neoepidermis at the wound margins were significantly decreased on day 6 in *P. aeruginosa*-infected wounds (Figure 8A,B). The infected wounds displayed a consistent distribution of PCNA-positive cells, indicating an early healing stage transitioning toward the proliferative phase. This was also observed following Def-GaPP treatment.

**Figure 9:**
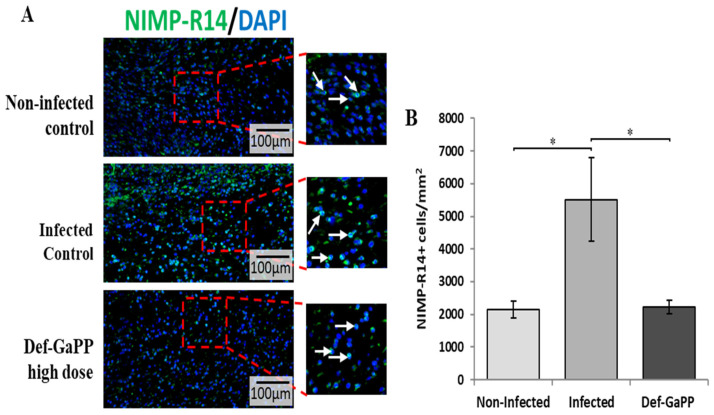
Analysis of neutrophil infiltration in a day-6 wound sample. (**A**) Representative fluorescence images of neutrophil cells in the wound (NIMP-R14 (green)/DAPI (blue)). (**B**) Corresponding analysis of neutrophil cells in the wound matrix. White arrows indicate NIMP-R14-positive cells. Data shown as mean ± SEM. * *p* < 0.05. Scale bar 100 μm, *n* = 8.

To further investigate the correlation between wound healing and inflammation, the wound sections were also stained for neutrophils and macrophages on the 6th day post-wounding. Studies to date have clearly demonstrated that delayed healing in chronic wound infection results in part from prolonged tissue inflammation [45,46]. In our study, the inflammatory response following the high-dose Def-GaPP treatment was investigated by analysing neutrophil infiltration within the wound bed and treatment effects on macrophage differentiation as an indication of the stage of healing. Neutrophil infiltration into the wound matrix was assessed as total number of NIMP-R14-positive cells (Figure 9A). At day 6, neutrophil infiltration into the wound bed was significantly increased in the infected control compared with the non-infected control. Importantly, Def-GaPP treatment of infected wounds significantly reduced neutrophil infiltration compared with the infected control, showing similar levels as the non-infected control (Figure 9B). Neutrophil infiltration is highest during the inflammatory phase of wound healing [44,47]. Lower neutrophil numbers in the Def-GaPP-treated wounds indicate that Def-GaPP reduced the inflammation caused by *P. aeruginosa* infection, and this agrees with previous studies that showed that topical Def treatment has strong anti-inflammatory and antioxidant properties in cutaneous wounds [48].

**Figure 10:**
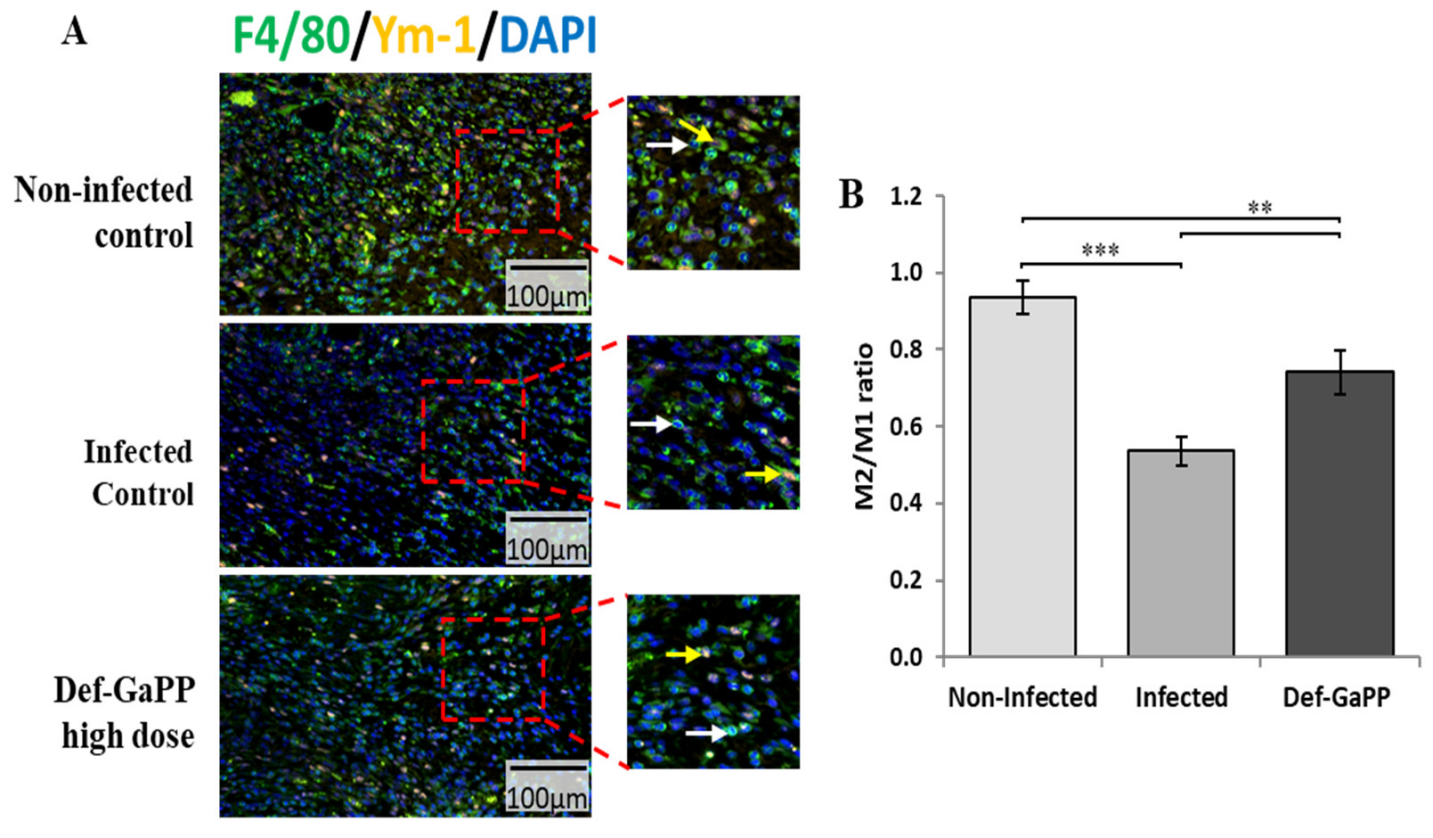
Analysis of macrophage cells in day-6 wound samples. (**A**) Representative fluorescence images of macrophage phenotypes in the wound (F4/80 (Green)/Ym-1 (orange)/DAPI (blue)). The white arrows indicate F4/80-positive M1 macrophages, and the orange arrows indicate co-stained F4/80-positive and Ym-1-positive M2 macrophages. (**B**) Corresponding ratio of M2/M1 macrophage phenotypes in the wound bed. Data shown as mean ± SEM. ** *p* < 0.01, *** *p* < 0.001. Scale bar 100 μm, *n* = 8.

To verify the function of Def-GaPP treatment in macrophage polarisation in vivo, immunofluorescence staining of M1 and M2 macrophages was analysed using F4/80 and Ym-1 markers to determine the anti-inflammatory M2/pro-inflammatory M1 ratio. Normally, the M1 macrophages would be the predominant macrophage phenotype at early stages of wound healing and in response to infection, while the M2 macrophages usually peak in later stages, around day 7 after injury, and increase in numbers once infection is cleared. Here, the representative images of F4/80/Ym-1 double-staining showed a high distribution of orange double-stained macrophages (yellow arrows) indicative of a high M2 macrophage phenotype count in response to Def-GaPP Chitogel treatment (Figure 10A). The quantitative analysis demonstrated a notably elevated M2/M1 ratio in the presence of Def-GaPP treatment compared with the infected control (Figure 10B). This indicates a significant anti-inflammatory effect of the Def-GaPP Chitogel treatment, contributing to the clearance of infection and acceleration of the wound healing process. As anticipated, the M2/M1 ratio was also higher in response to the non-infected control due to the absence of infection and a similar wound healing profile to the treated infected groups. Meanwhile, the level of M2/M1 was the lowest for the untreated infected groups, suggesting the presence of inflammation and delayed healing.

The decreased M2/M1 ratio observed in the infected control indicates increased inflammation compared with the non-infected control, demonstrating a higher presence of pro-inflammatory M1 macrophages in the infected control-treated wounds. High-dose Def-GaPP Chitogel treatment, while still being lower than the non-infected control group, significantly increased the M2/M1 ratio compared with the infected control, indicating reduced tissue inflammation following Def-GaPP treatment (Figure 10A,B). Collectively, these data indicate that the high-dose Def-GaPP treatment has a positive impact on wound healing by clearing the infection and reducing tissue inflammation. The ability of Def-GaPP to clear infection is crucial for creating a conducive environment for healing, while the reduction in inflammation signifies a balanced immune response that is essential for progressing through subsequent phases of the wound healing process.

## 4. Conclusions

In this investigation, the combined approach of incorporating Def-GaPP agents into a biocompatible Chitogel delivery system demonstrated a dose-dependent efficacy in diminishing the bacterial load associated with *P. aeruginosa* chronic biofilm wound infection. The in vitro antibacterial tests showed that the Def-GaPP Chitogel high-dose treatment significantly reduced biofilms and decreased bacterial attachment. The treatment showed positive effects on cellular proliferation and migration, while being biocompatible against skin cells and showing no evidence of the mammalian cell toxicity often observed with many antimicrobial treatments. Preclinical in vivo studies further validated the antibacterial properties of Def-GaPP treatment, illustrating >1 log reduction in bacterial load, equivalent to 90% bacterial reduction and a significantly decreased wound biofilm biomass comparable to that of antibiotic standard controls. Importantly, the positive effects of Def-GaPP antimicrobial treatment were not accompanied by the impairments in healing often observed with topical antimicrobial treatments. Unlike the antibiotic treatment control, the high-dose Def-GaPP treatment did not impair the rate of wound closure, demonstrating significantly decreased neutrophil infiltration and promoting anti-inflammatory M2 macrophage phenotypes within the wound bed. We acknowledge the limitations of the short preclinical study and suggest that future research should examine the antimicrobial efficacy and wound-healing effects of high-dose Def-GaPP using porcine infected wound models to obtain a deeper understanding of infection clearance, complete healing, and potential effects on tissue scarring and regeneration. Overall, this study demonstrates that optimal dosage of Def-GaPP Chitogel is a promising antimicrobial treatment strategy for *P. aeruginosa* infection, offering an innovative solution for improved management of clinical wounds.

## Data Availability

Data available on request from co-authors.

## Supplementary Materials

The following supporting information can be downloaded at https://www.mdpi.com/article/10.3390/ma17040793/s1, Table S1: Primary antibodies; Table S2: Secondary antibodies; Table S3: Minimum inhibitory concentration of liquid Def and GaPP treatment on *P. aeruginosa* (*n* = 3); Table S4: Zone of inhibition.
