# Supplementary material for: Deferiprone-Gallium-Protoporphyrin Chitogel Decreases Pseudomonas aeruginosa Biofilm Infection without Impairing Wound Healing
Source: Materials (Basel). 2024 Feb 7;17(4):793. doi: 10.3390/ma17040793 (PMC10889926; doi:10.3390/ma17040793)
Supplement: Supplementary file 1 [file materials-17-00793-s001.zip › materials-2804359-supplementary.pdf]

Supplementary

Table S1: Primary antibodies

Antibody	Supplier	Working concentration
Rat anti-NIMP-R14 #sc-59338	Santa Cruz Biotechnology	0.5µg/mL
Mouse anti-PCNA #sc-56	Santa Cruz Biotechnology	1µg/mL
Rat anti-F4/80 #MCA497R	Bio-Rad Laboratories	5µg/mL
Rabbit anti-YM-1 #60130	Stem Cell Technologies	0.37µg/mL
Mouse anti-Caspase-1 #sc-56036	Santa Cruz Biotechnology	1µg/mL
Mouse anti-Annexin-V #sc-74438	Santa Cruz Biotechnology	1µg/mL
Rabbit anti-Collogen 1 #600-401-103-0.5	Rockland	5µg/mL
Rabbit anti-Collogen 3 #600-401-105-0.5	Rockland	5µg/mL

Table S2: Secondary antibodies

Antibody	Supplier	Working concentration
Alexa Flour goat anti-rat 488 #A11006	Invitrogen	5µg/mL
Alexa Flour goat anti-mouse 568 #A11004	Invitrogen	5µg/mL
Alexa Flour goat anti-rabbit 568 #A11011	Invitrogen	5µg/mL
Alexa Flour goat anti-rabbit 488 #A27034	Invitrogen	5µg/mL

Table S3. Minimum inhibitory concentration of liquid Def and GaPP treatment on *P. aeruginosa.* n=3.

	Def (mM)	GaPP (µg/ml)	Def-GaPP (mM - µg/ml)
P. aeruginosa	10	500	2.5 - 125

Table S4. Zone of inhibition of *P. aeruginosa* treated with Def and GaPP Chitogels bacteria were grown on tryptic soy agar plates incubated overnight and with either Chitogel, Def 10mM, GaPP 500µg/mL or Def-GaPP 10mM-500µg/mL treatment. Results displayed as Mean (mm) *** p<0.001 compared to Chitogel; ^^^p<0.001 compared to Def-GaPP. n=6.

	Chitogel (mm)	Def (mm)	GaPP (mm)	Def-GaPP (mm)
P. aeruginosa	0.0 ± 0.0	10.8 ± 0.1***^^^	0.0 ± 0.0^^^	20.3 ± 0.1***
